# Editorial: Neuropsychology of human social decision-making: the role of emotions

**DOI:** 10.3389/fnins.2024.1405057

**Published:** 2024-06-03

**Authors:** X. T. Wang, Sarah Hill, Hongjian Cao

**Affiliations:** ^1^Applied Psychology Division of the School of Humanities and Social Science, The Chinese University of Hong Kong, Shenzhen, China; ^2^Psychology Department, Texas Christian University, Fort Worth, TX, United States; ^3^Psychology Department, The University of Hong Kong, Pokfulam, Hong Kong SAR, China

**Keywords:** neuropsychology, social decision making, group decision, emotions, descriptive vs. experiential decisions, interpersonal decision-making

As social beings, humans live in an increasingly complex social environment, particularly in the current time of the Internet and AI. Characterized by volatility, uncertainty, complexity, and ambiguity (VUCA), decision-making in such an environment has long been considered among the cores of human social behaviors. Notably, rapid advancements in neuroscientific techniques in the past few decades allowed a more comprehensive approach to understanding cognitive as well as emotional mechanisms underlying social decision-making (see Ernst and Paulus, 2005; Rilling and Sanfey, 2011; Ruff and Fehr, 2014; Suzuki and O'Doherty, 2020; Wallace and Hofmann, 2021).

Against this backdrop, this Research Topic represents a collective effort to follow such research leads. We gathered a group of scholarly works on the neuropsychology of human social decision-making. First, in their conceptual article, Crivelli and Balconi called for more attention to the emergence of shared representations, affective experience, and intentions shaping group relational dynamics. They proposed a multi-agent perspective on shared affects and interpersonal syntonization in group decision-making. In addition, three empirical studies were also selected. By instructing observers to voluntarily identify the orientation of a Necker cube while manipulating its ambiguity, Kuc et al. studied the perceptual bias in favor of the from-above Necker cube perspective in goal-directed behavior. In addition, they also analyzed electroencephalogram (EEG) signals to identify potential biomarkers that could explain the observed perceptual bias. Using the trust game as the decision-making paradigm, Zhu et al. conducted two experiments to examine trust asymmetry from the perspective of risk sources under descriptive vs. experiential decisions. Last but not least, in two studies using behavioral experimental procedures and EEG techniques, Vicente et al. investigated the neurophysiological correlates of interpersonal discrepancy and social adjustment in an interactive decision-making task in dyads.

While addressing different neural aspects of social decision-making, we hoped to sort out the roles of emotions in regulating social decisions. This still unfulfilled objective leaves us with more questions for future research. Over the years, the views of emotions in decision-making have undergone several transformations. The role of emotions in decision-making changed from a disturbing factor in utility theories of neoclassic economics (Elster, 1998) to a rich resource of information (Clore and Huntsinger, 2007) and the key to success (Goleman, 2006). While the Somatic Marker Hypothesis (Damasio, 1996) highlights the inevitability of emotions in decision-making, the Risk as Feelings Hypothesis (Loewenstein et al., 2001) emphasizes the unique role of anticipatory emotion (as opposed to reactive emotions) induced by episodic future thinking for guiding rational decisions (Wang et al., 2022). The articles in this Research Topic also provide insightful discussions on future directions for the neuroscience of emotions in social decision-making.

## Author contributions

XW: Writing – original draft, Writing – review & editing. SH: Writing – review & editing. HC: Writing – original draft, Writing – review & editing.
